# Using predictive machine learning models for drug response simulation by calibrating patient-specific pathway signatures

**DOI:** 10.1038/s41540-021-00199-1

**Published:** 2021-10-27

**Authors:** Sepehr Golriz Khatami, Sarah Mubeen, Vinay Srinivas Bharadhwaj, Alpha Tom Kodamullil, Martin Hofmann-Apitius, Daniel Domingo-Fernández

**Affiliations:** 1 Department of Bioinformatics, Fraunhofer Institute for Algorithms and Scientific Computing, Sankt Augustin, 53757 Germany; 2 Bonn-Aachen International Center for Information Technology (B-IT), University of Bonn, 53115 Bonn, Germany; 3 Fraunhofer Center for Machine Learning, Sankt Augustin, Germany; 4 Enveda Biosciences, Boulder, CO 80301 USA

**Keywords:** Computer science, Computer modelling, Software, Drug discovery

## Abstract

The utility of pathway signatures lies in their capability to determine whether a specific pathway or biological process is dysregulated in a given patient. These signatures have been widely used in machine learning (ML) methods for a variety of applications including precision medicine, drug repurposing, and drug discovery. In this work, we leverage highly predictive ML models for drug response simulation in individual patients by calibrating the pathway activity scores of disease samples. Using these ML models and an intuitive scoring algorithm to modify the signatures of patients, we evaluate whether a given sample that was formerly classified as diseased, could be predicted as normal following drug treatment simulation. We then use this technique as a proxy for the identification of potential drug candidates. Furthermore, we demonstrate the ability of our methodology to successfully identify approved and clinically investigated drugs for four different cancers, outperforming six comparable state-of-the-art methods. We also show how this approach can deconvolute a drugs’ mechanism of action and propose combination therapies. Taken together, our methodology could be promising to support clinical decision-making in personalized medicine by simulating a drugs’ effect on a given patient.

## Introduction

Applying machine learning (ML) methods to biomedical data has enormous potential for the development of personalized therapies,^1^ drug repurposing,^2^ and drug discovery.^3^ The data exploited by these methods can comprise multiple modalities including imaging data,^4^ chemical structure information,^5^ and natural language data.^6^ However, the widespread availability of transcriptomics data (e.g., RNA-Sequencing (RNA-Seq), microarrays, etc.) along with its capacity to provide a comprehensive overview of biological systems have made this particular modality a popular choice for various computational methods. Although this modality can reveal both molecular signatures as well as phenotypic changes that occur in altered states, pathway analyses are often performed to map measured transcripts to the pathway level due to high dimensionality and correlations present in transcriptomics datasets.^7,8^ This transformation facilitates the training of ML/AI models by reducing dimensional complexity whilst enhancing interpretive power.^9^ However, such a transformation implicates the use of prior pathway knowledge^10^ from databases such as KEGG^11^ and Reactome.^12,13^

The transformation of data from the transcriptomics to the pathway level can be used to generate pathway features (i.e., sets of genes involved in a given pathway that are coordinately up or down-regulated), the latter of which have broad applications in drug discovery and drug response prediction.^14^ For instance,^15–17^ exploited the concept of anti-similarity between drugs and disease-specific pathway signatures to identify therapeutic candidate drugs that can potentially revert disease pathophysiology. Furthermore,^18^ shows how pathway signatures derived from cell lines using kernelized Bayesian matrix factorization can be used for drug response prediction.

Alternatively, other methods can generate individualized pathway features from a population of patients or cell lines.^19^ These features, or pathway activity scores, can subsequently be used for several downstream ML applications including classification tasks and survival prediction.^8,20^ In addition,^21^ showed how ML models can be used to predict drug response using pathway activity scores derived from cell lines. Furthermore, another example from^22^ demonstrated how modeling individualized pathway activity scores from Fanconi anemia patients can reveal potential targets for therapeutic interventions. Finally, similar approaches have been used to prioritize drug treatments in the cancer context.^23,24^

While these methods have shown how pathway signatures can be used for drug discovery and drug response prediction, existing methods thus far fail to account for two important factors. First, as the response triggered by a drug in a given patient may differ if administered in another, these methods should account for patient heterogeneity which is crucial in designing individualized therapies. Second, specific indications may be improved or corrected by a drug combination approach or through the administration of multi-target drugs.

In this work, we present an intuitive methodology that exploits the predictive power of ML models to simulate drug response by calibrating pathway signatures of patients. We first trained an ML model (i.e., elastic net penalized logistic regression model) to discriminate between disease samples and controls based on sample-specific pathway activity scores. Next, we simulate drug responses in patients using a scoring algorithm that modifies a patient’s pathway signatures using existing knowledge on drug-target interactions. We hypothesize that promising drug candidates for a given condition would modify pathway activity scores of patients in such a way that they closely resemble scores of controls. Thus, using the previously trained ML model, we then evaluate whether patients with modified pathway scores are now classified as normal as a proxy for promising drug candidates. We demonstrate the scalability and generalizability of our methodology by simulating over one thousand drugs from two independent drug-target datasets on four cancer indications. Furthermore, we show how our methodology is able to recover a large proportion of clinically investigated drugs on these four indications, outperforming six comparable state-of-the-art methods. Finally, we show how the most relevant pathways identified by our methodology can be used to better understand the biology pertaining to a given condition.

## Results

**Fig. 1:**
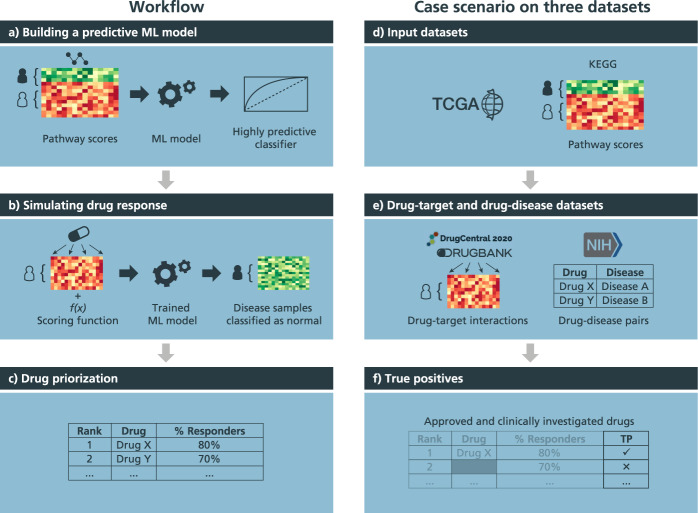
Conceptual overview of the drug simulation workflow and case scenario on multiple datasets. (**a**) Pathway activity scores are used to train a highly predictive ML model that differentiates between normal and disease samples, labeled green and red on the heatmap, respectively. (**b**) Next, pathway scores of disease samples are modified by using drug-target information and applying a scoring algorithm that simulates the effect of a given drug at the pathway-level. Using the modified pathway scores of disease samples, the trained ML classifier is then used to evaluate whether these modified disease samples that were previously classified as “diseased” could now be classified as “normal”. (**c**) Finally, we use the proportion of disease samples now classified as normal (i.e., % responders) as a proxy to identify candidate drugs and propose combination therapies. (**d**) To demonstrate the methodology in a case scenario, we first performed ssGSEA using pathways from KEGG and the BRCA, LIHC, PRAD, and KIRC TCGA datasets to acquire sample-wise pathway activity scores. (**e**) Next, we obtained known drug-target interactions from DrugBank and DrugCentral and drug-disease pairs (i.e., FDA-approved drugs and drugs under clinical trials for a given condition) from Clinicaltrials.gov and FDA-approved drugs, of which, the latter two were used as a ground-truth list of true positives (TP). (**f**) To simulate drug treatments of patients from the aforementioned TCGA datasets using their pathway activity scores (i.e., Fig. 1d), we applied the methodology described in Fig. 1a–c to acquire a ranking of drugs based on the proportion of disease samples that were treated. Finally, we identified the proportion of drugs ranked by our methodology that were true positives for the four TCGA datasets and compared this proportion to random chance.

We present a workflow designed to approximate a drug’s effect on a patient by intentional modifications to patient-specific features, specifically, pathway activity scores, by employing highly predictive ML models trained to differentiate between normal and disease samples (Fig. 1). In the first subsection, we validate our approach by (i) evaluating its capability in retrieving FDA-approved drugs and those in clinical trials for multiple cancer datasets and, (ii) comparing the results yielded by our approach against several equivalent methods. Then, in the following two subsections, we investigate the drug candidates prioritized by our approach and the specific pathways targeted by these prioritized drugs, respectively. Finally, we show the utility of our approach in predicting the effects of a combination of drugs for applications in combination therapy and for the identification of potential adverse events associated with drug combinations.

### Validation of the methodology and comparison against equivalent approaches

In this subsection, we investigate the drug candidates prioritized by our methodology in four different cancers and evaluate the ability of our approach to identify approved and clinically investigated drugs (i.e., true positives). Table 1 shows that only a minority of the drugs present in both drug-target datasets were prioritized by our methodology given that a stringent threshold was employed which required that prioritized drugs change the predictions of at least 80% of the patients (see “Materials and Methods” and Supplementary Figs. 7, and 8 for details on the selection of this threshold). Overall, our methodology is able to recover a large proportion of true positives (ranging from 13% to 32%) in all four cancers as well as in both drug-target datasets (Table 1). This wide range may be attributable to a disproportion in the number of true positives that exist for each of the cancer datasets (e.g., BRCA has more than twice as many FDA-approved drugs and drugs in clinical trials than LIHC) as well as to the size of the drug-target datasets (i.e., DrugBank contains twice as many drugs as DrugCentral).

**Table 1:** Number of FDA-approved and clinically tested drugs recovered for both drug-target datasets (i.e., DrugBank (DB) and DrugCentral (DC)) across the four investigated cancers.

Dataset	DB Prioritized	DB Approved (total)	DB Clinical trials (total)	DB Proportion of true positives (%)	DC Prioritized	DC Approved (total)	DC Clinical trials (total)	DC Proportion of true positives (%)
BRCA	129	8 (26)	23 (182)	31/129 (24.03%)	19	2 (14)	4 (115)	6/19 (31.57%)
LIHC	74	2 (5)	11 (50)	13/74 (17.56%)	19	1 (1)	2 (35)	3/19 (15.78%)
PRAD	68	2 (13)	18 (134)	20/68 (29.41%)	19	1 (7)	3 (84)	4/19 (21.05%)
KIRC	88	2 (8)	10 (44)	12/88 (13.63%)	26	3 (3)	2 (25)	5/26 (19.2%)

In the first column for each drug-target dataset (“Prioritized”), we report the number of drugs that changed the predictions for at least 80% of the patients for each cancer type. The second column (“Approved”) reports the number of FDA-approved drugs among these prioritized drugs as well as the total number of FDA-approved/clinically tested drugs present in each dataset between parentheses. Similarly, the third column (“Clinical trials”) reports the number of drugs tested in clinical trials among the prioritized drugs and the total number of FDA-approved/clinically tested drugs between parentheses. Finally, the last column (“Proportion of true positives”) reports the proportion of true positives (both FDA-approved and clinically tested drugs) among the prioritized drugs.

As a comparison, the methodology proposed by^25^ reported lower proportions of true positives than our approach for the BRCA and PRAD datasets with 21.42% and 15.94%, respectively (Supplementary Table 1). Furthermore, four additional methods present that were benchmarked by^25^ yielded even lower results on the same two cancer datasets (Supplementary Tables 2–8). Similarly,^26^ also reported a lower proportion of true positives than our approach for the BRCA and PRAD datasets with 0.8% and 0.4%, respectively (Supplementary Table 9). Overall, the performance across all six methods varied from 0% to 11.53% for BRCA, and from 0.50% to 22.22% for PRAD and is summarized in Supplementary Table 10.

In addition, the proportion of true positives yielded by our methodology is significantly higher than what one would expect by chance (see “Materials and Methods”). Furthermore, we compared the number of prioritized drugs found in the original DrugBank and DrugCentral datasets to the number of prioritized drugs obtained in the robustness experiments in which we applied our methodology to drugs with randomly generated targets and target interactions (Supplementary Fig. 1). We found that all permutation experiments yielded a significantly lower number of prioritized drugs. Because our methodology can capture a much greater number of prioritized drugs on a real dataset, this validation highlights the capability of our approach to prioritize drugs with targets in relevant pathways that are key to change the predictions of patients.

As a final remark, we explored the performance of our methodology when varying one of the weights while keeping the other two constant to better understand how sensitive the results are to the selected weights **(**Supplementary Tables 11, 12). We have observed that the proportions of true positives recovered mainly vary between 15% and 35% in the three test disease datasets for both drug-target datasets when *W*_*1*_ (i.e., the weight assigned to the quartile that contains the most dysregulated pathways) is in the range of 10–20. There are multiple cases where we found sets of weights yielding better results than the ones presented in Table 1 if exclusively looking at a single or two specific disease datasets (Supplementary Table 13). In contrast, we observed that when weights are low (e.g., *W*_*1*_ = 1), our approach often does not yield any prioritized drugs (Supplementary Table 14), as in these cases, the modified pathway activity scores are not sufficient enough to change the predictions of the ML model.

### In-depth investigation of the prioritized candidate drugs

Apart from the previous quantitative evaluation of our methodology, we conducted an in-depth analysis of the prioritized drugs to better understand the predictions made by our approach. Below, we focus on drugs prioritized using the DrugCentral dataset as this dataset contains a fewer number of prioritized drugs than DrugBank.

**Fig. 2:**
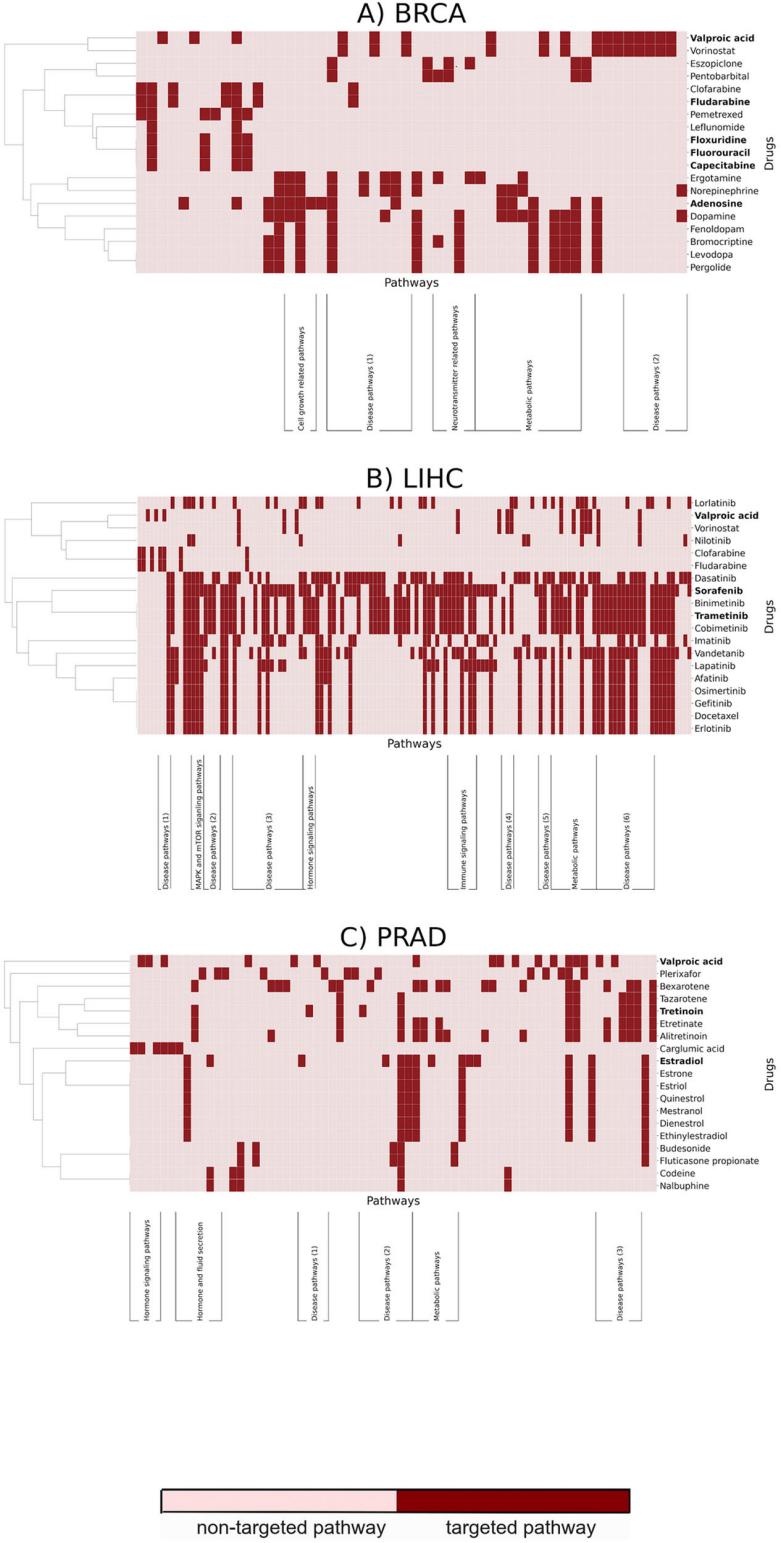
Pathways targeted by prioritized drugs in DrugCentral for each of the three cancer test datasets. The X axis corresponds to pathways targeted by any of the prioritized drugs (i.e., pathways not targeted by any prioritized drug are omitted for better visualization). Prioritized drugs for each cancer dataset have been clustered based on the pathways they target and are reported on the Y axis. Of the prioritized drugs, those that correspond to true positives are highlighted in bold. If a set of three or more similar pathways was clustered together, we manually assigned these pathways into distinct classes (Y axis) Pathway names and cluster information are available as a Supplementary File and the equivalent figures for DrugBank are available as Supplementary Figs. 2–4.

In the breast cancer dataset (BRCA), we identified a major class of drugs based on their mechanisms of action (Fig. 2a). This class targeted DNA and RNA metabolism and included commonly used anti-tumor drugs. One example of this group of drugs is fluorouracil, which targets thymidylate synthase, thereby inhibiting the formation of thymidylate from uracil.^27^ This drug is a chemotherapy medication commonly used to treat several cancers.

In the prostate cancer dataset (PRAD), we found that the majority of drugs were related to hormone metabolism and regulation (Fig. 2c). Due to the key role of sex steroid hormones in its initiation and progression,^26^ this cancer is classified as hormone-dependent. Thus, current treatments are often directly targeted towards these hormones, such as androgen deprivation therapy, which represents the major therapeutic option for treatment of advanced stages of this cancer.^28–30^

The third dataset, LIHC, corresponds to hepatocarcinoma. Interestingly, the vast majority of the candidate drugs in this dataset (14/19) are tyrosine kinase inhibitors (TKI) corresponding to anti-tumor drugs already FDA-approved for other cancers^31^ (Fig. 2b). Since these kinases act as regulatory players in several cancer signaling pathways that can be hyperactivated, TKIs are used to “switch-off” these pathways, indirectly inhibiting cell growth.^32^ One of the predicted drugs is sorafenib, which was the first TKI to be approved for the treatment of liver carcinoma and still remains as a first-line therapy. Similarly, another predicted drug, trametinib, is a dual-kinase inhibitor that is used in the treatment of advanced liver cancer. Finally, two of the remaining non-TKIs are also employed as chemotherapy drugs as they inhibit the synthesis of nucleotides.

### Investigation of pathways targeted by the prioritized drugs

Here, we interpret and analyze the results yielded by our methodology for multiple datasets by investigating the pathways targeted by the drugs prioritized through our approach. We identified clusters of pathways belonging to several distinct classes (Fig. 2). Not surprisingly, we found that various metabolic pathways appeared in all three test datasets as the regulation of metabolism plays an important role in numerous cancers. Given that each of the three test datasets were cancer subtypes, intuitively, we also observed several disease-relevant pathways targeted by the prioritized drugs, among which were ~30 cancer-related pathways from KEGG (e.g., prostate cancer, pancreatic cancer, bladder cancer, and breast cancer).

Drugs that were prioritized by our approach (Fig. 2) were likewise clustered based on the pathways they targeted to assess whether drugs that targeted the same pathway fell within the same class of drugs. Prioritized drugs for liver cancer could be clustered into four different classes of tyrosine kinase inhibitors: (i) JAK inhibitors (i.e., sorafenib, vandetanib, erlotinib, and lapatinib), (ii) ALK inhibitors (i.e., lorlatinib), (iii) BCR–Abl (i.e., nilotinib, dasatinib, and imatinib), and (iv) and EGFR inhibitors (i.e., afatinib).^33^ In addition, we found MEK kinase inhibitors, specifically trametinib and cobimetinib. Finally, we found that while some drugs were able to change the predictions by targeting only a limited number of pathways (e.g., fludarabine in breast cancer and liver cancer), other drugs could change predictions by targeting several pathways (e.g., tretinoin in prostate cancer and trametinib in liver cancer).

Among the most commonly targeted pathways by the prioritized drugs in liver carcinoma, we found Ras/Raf/MAPK and PI3K/AKT/mTOR signaling, both of which have been reported to play important roles in the development of this type of cancer.^34^ One of the prioritized drugs, sorafenib, is a multi-kinase inhibitor that targets several kinases including RFA1, PDGFR, and FLT3, which are involved in both tumor proliferation and angiogenesis.^35,36^ Sorafenib has been shown to inhibit tumor cell proliferation by blocking the Ras/Raf/MAPK pathway and to inhibit angiogenesis by blocking PDGFR signaling^37^ (Supplementary Table 15).

### Prioritizing combination therapies

Combination therapies are widely used for treating indications like cancer as they can often lead to the inhibition of the compensatory signaling pathways that maintain the growth and survival of tumor cells. Here, we demonstrate how our methodology can be extended to predict the effects of a combination of drugs. To reduce the computational complexity associated with running our methodology on all possible combinations of drug pairs from both drug-target datasets (i.e., DrugBank and DrugCentral), we exclusively applied our method on all possible pairs from the set of prioritized drugs. Table 2 lists a subset of combinations of prioritized drugs, alongside the proportion of patients that they reclassify as normal (i.e., proportion of treated patients).

**Table 2:** Examples of predicted combination therapies.

Cancer type	Drug 1	Drug 2	Proportion of responders (%)	Reference
Liver cancer	Sorafenib	Trametinib	87%	^53^
Liver cancer	Erlotinib	Sorafenib	87%	^54^
Breast cancer	Vorinostat	Capecitabine	88%	^55^

For two of the three test datasets (i.e, LIHC and PRAD), nearly all drug pairs yielded better results (i.e., larger proportion of disease samples predicted as normal) than the use of a single drug alone. In the BRCA dataset, however, multiple combinations yielded worse results than those observed with single drug therapy. For example, the combination of bromocriptine with valproic acid decreased the proportion of treated patients from 80% to <10%. Specifically, bromocriptine is an adrenergic receptor agonist that stimulates the beta-adrenergic signaling pathway, which in turn prompts tumor angiogenesis and cancer development.^38^ Similarly, valproic acid is a histone deacetylase which also induces beta-adrenergic signaling, thus promoting cancer progression.^39^ Therefore, the combination of these two drugs not only fails to treat the cancer, but may in fact lead to the worsening of the condition.

## Discussion

Here, we have presented a powerful machine learning framework to simulate drug responses for applications in drug discovery and precision medicine. We demonstrate our methodology on four different cancer datasets and two independent drug-target datasets by using patient-specific pathway signatures to train highly predictive models which we use as a proxy for drug candidate identification. Across all datasets, our results yielded a larger proportion of FDA-approved drugs as well as drugs investigated in clinical trials than six comparable approaches for the indications we studied, suggesting that other drugs prioritized by our methodology may also represent promising candidates for repurposing. In addition, in contrast to the other methodologies, our approach is able to prioritize drugs for individual patients, making it suitable for precision medicine applications. Finally, we also show how our methodology can be applied to propose drug combinations as well as to reveal sets of dysregulated pathways that could be used as possible targets.

Currently, there exist several limitations to this study; first, although our scoring algorithm used to simulate drug response has been shown to yield promising results in the four datasets analyzed, other scoring algorithms may be better suited for different datasets and/or applications. For instance, we could tailor the current scoring algorithm for drug discovery to learn pathway signatures from approved drugs and use these drugs to prioritize candidates that exhibit similar patterns of activity. Second, although we recommend the selection of weights following a similar logic to the one we have presented here (i.e., assigning larger weights to the quartile containing the most dysregulated pathways and lower weights for others), it may be the case that weights must be tuned for other datasets to yield promising candidates. Third, since our methodology relies on pathway signatures derived from transcriptomics data, it is inherently limited to indications where this modality is highly predictive. In other words, pathway activity scores must be readily separable between disease and normal samples in the disease we investigate as we require highly predictive models that can guarantee the change in the predicted class label is exclusively caused by the drug simulation step and not by the lack of accuracy of the model. Thus, it would be less effective in indications where transcriptomics have limited prediction power to discriminate between normal and disease samples, such as Parkinson’s disease.^40^ Finally, while we have demonstrated our approach with a commonly used sample-wise enrichment method, ssGSEA does not take network topology into consideration. Thus, in the future, other enrichment methods that leverage the topological information of pathways can be used to generate the pathway activity scores used by our algorithm.

Beyond this proof-of-concept, our methodology can be extended to include several additional functionalities. For instance, drug administration could be simulated in an ML model that takes into consideration temporal dimensions (e.g., event-based models,^41^ survival analysis^42^). Furthermore, in this paper we trained a simple ML model, nonetheless, the same strategy could be applied to more complex ML or AI models. Since the elastic net penalty encourages sparsity, one may also use the coefficients of an ML model as a preliminary method of filtering for significant features. To save time, the total set of drug candidates can be subset to only those which directly affect the features that significantly affect the prediction capabilities of the model. In addition, we restricted our analysis to a single pathway database as it was sufficient to deploy a predictive ML model for the specific classification task we presented. However, by incorporating pathway information from other databases into the ML model, we can increase the total number and coverage of pathways to potentially reveal additional pathway targets. Similarly, the use of different drug-target databases such as ExCAPE-DB^43^ could broaden the chemical space and lead to the identification of new candidates. By combining brute-force and reverse engineering approaches, one can also identify the most effective pathway scores a drug should target for any given indication; thus, tailoring the presented methodology towards drug discovery. Finally, due to limited data for all possible responses a given patient could have to a particular drug in large cohorts, we relied upon classic drug repurposing validation strategies to demonstrate the efficacy of our approach. However, with enough training data, our methodology could be deployed to support clinical decision-making in personalized medicine by simulating the effect of drugs on individual patients.

## Materials and methods

The initial step of our methodology consists of generating patient-specific features that can be used for model training. Although in this work, we employed pathway activity scores (see subsection “Calculating individualized pathway activity scores”), other features could also be used for the same purpose. Using these scores, we trained an ML model (subsection “Building a predictive classifier”) that can accurately discriminate between sample classes (e.g., disease vs normal). Next, we developed a scoring algorithm aimed to simulate the effect of a drug intervention at the pathway-level by modifying the pathway activity scores of disease samples (subsection “Scoring algorithm”). Then, the method uses the modified pathway activity scores as an input in the trained model to assess whether samples that were previously classified as “diseased” could now be classified as “normal” as a proxy for drug candidates (Subsection “Drug response prediction and prioritization”). Then we validate and evaluate our approach by presenting the datasets used for our case scenario and comparing our methodology against six equivalent approaches. Finally, we provide details on the implementation.

### Datasets

Datasets from The Cancer Genome Atlas (TCGA)^44^ were retrieved from the Genomic Data Commons (GDC; https://gdc.cancer.gov) portal through the R/Bioconductor package, TCGAbiolinks (version 2.16.3;^45^) on 04-08-2020 (Fig. 1d). Gene expression data from RNA-Seq was quantified using the HTSeq and raw read counts were normalized using Fragments Per Kilobase of transcript per Million mapped reads upper quartile (FPKM-UQ). Gene identifiers were mapped to HUGO Gene Nomenclature Committee (HGNC) symbols where possible. The datasets downloaded include The Cancer Genome Atlas Breast Invasive Carcinoma (TCGA-BRCA), The Cancer Genome Atlas Prostate Adenocarcinoma (TCGA-PRAD), The Cancer Genome Atlas Liver Hepatocellular Carcinoma (TCGA-LIHC), and The Cancer Genome Atlas Kidney Renal Clear Cell Carcinoma (TCGA-KIRC) (Supplementary Table 16). We would like to note that due to the design of our methodology, we required the datasets to have a large sample size to conduct the hyperparameter optimization of the ML model and the cross validation strategy described below.

### Calculating individualized pathway activity scores

We used single-sample GSEA (ssGSEA),^46^ a commonly used tool to generate patient-specific pathway activity scores. Normalized gene expression (FPKM-UQ) and pathway definitions (i.e., gene sets) were provided as input and were converted to scores through ssGSEA (Supplementary Table 17; Supplementary Fig. 5). As a reference database, we used 337 pathways from KEGG (downloaded on 01-04-2020) as it is the most widely used pathway database and a standard for the most commonly used pathway activity scoring methods^18^ (Fig. 1d).

### Building a predictive classifier

Patient-specific pathway activity scores generated by ssGSEA were used to generate a ML classifier to distinguish between normal and tumor sample labels for each of the four datasets. The classification was conducted using an elastic net penalized logistic regression model^47^ as regularized models have been shown to be generally well suited for -omics data which typically contains a disproportionate number of features to samples, and specifically well suited for these datasets.^21^ Furthermore, we previously used this ML model on the same TCGA datasets,^19^ yielding AUC-ROC and AUC-PR values close to 1 (Supplementary Fig. 6), in line with Mubeen et al. (2019). Prediction performance was evaluated via 10 times repeated 10-fold stratified cross-validation and tuning of elastic net hyper-parameters (i.e., *l*_1_, *l*_2_ regularization parameters) via grid search was performed within the cross-validation loop to avoid over-optimism.^48^

### Scoring algorithm

To modify the pathway activity scores for disease samples, we developed a scoring algorithm to replicate the effect of a drug at the pathway-level. The scoring algorithm exploits interactions from drug-target datasets to modify the activity scores of pathways containing the target(s) of a drug (see example in Supplementary Fig. 10). We describe the scoring algorithm in Box 1.

For each drug-pathway association, the pathway is assigned an effect score *ES* which is equivalent to a drug’s effect on a protein target coming from drug-target datasets (i.e., activation and inhibition relationships given +1 and −1 labels, respectively). For pathways that contain multiple protein targets, the ES is equivalent to the mean of these effects (e.g., if a drug activates a protein in a pathway but also inhibits a second protein in the same pathway, the overall effect of the drug on the pathway (ES) would be 0). The absolute values of the mean differences between healthy and disease groups are calculated for each pathway μ_H-D_*(p)* while their quartiles are then computed on line 2. Then, from lines 3–12, for each disease sample, if the *ES* of a pathway *p* is less than or greater than 0, the scoring algorithm calculates a calibration score *CS* as the product of the absolute value of the original pathway activity score *PAS*, the weight *w*, and the effect of the drug on the pathway *sgn(p)* (i.e., −1, 0 or 1). We assign *w* based on the quartile μ_H-D_*(p)* pathway *p* falls into. For pathways with larger mean differences between groups, weights are assigned greater values, while pathways with smaller differences are weighted less (see example in Supplementary Text 1). On lines 13–14, if the *ES* of a pathway *p* is 0, the *CS* is assigned the value of the original *PAS*. Finally, on line 15, the *CS* is returned as a score that simulates the effect of a drug on a pathway for a disease sample.

Box 1 **Scoring algorithm pseudocode**. The pseudocode outlines the scoring algorithm used to modify the pathway activity scores of a given patient **Scoring Algorithm**

**Require:**

    Set of pathways containing the protein target(s) of the drug, $\left\{ {P|p \in P} \right\}$

    Set of samples, $\left\{ {S|s \in S} \right\}$

    Set of healthy and disease samples, $\left\{ {H,D|H,D \in S|\forall h \in H,d \in D} \right\}$

    Set of target labels, $\left\{ {T|t \in T} \right\}$

    Array consisting of effect scores for all pathways,

          

$$\left\{ {{{{{{\mathrm{ES}}}}}}|{{{{{\mathrm{ES}}}}}}\left( p \right) \in {{{{{\mathrm{ES}}}}}},{{{{{\mathrm{ES}}}}}}\left( p \right) = \frac{1}{N}\mathop {\sum }\limits_{j = 0}^N t_j\left( p \right)} \right\}$$

    Where, *N* is the number of targets that are affected by a drug in pathway *p*

    Matrix consisting of original pathway activity scores for disease samples, ${{{{{\mathrm{PAS}}}}}}$

    Array consisting of the absolute values of mean differences between sample groups for each $p$, $\mu _{{{{{{\mathrm{H}}}}}} - {{{{{\mathrm{D}}}}}}} = \left| {\mu _{{{{{\mathrm{H}}}}}} - \mu _{{{{{\mathrm{D}}}}}}} \right|$

 **1:**  **function** SCORING_FUNCTION $$\left( {D,P,{{{{{\mathrm{ES}}}}}},{{{{{\mathrm{PAS}}}}}},\mu _{{{{{{\mathrm{H}}}}}} - {{{{{\mathrm{D}}}}}}}} \right)$$

 **2:**   Compute quartiles, $Q_1,Q_2,Q_3$, for all values of $\mu _{{{{{{\mathrm{H}}}}}} - {{{{{\mathrm{D}}}}}}}$

 **3:**   **for all** $d \in D$ **do**

 **4:**    **for all** $p \in P$ **do**

 **5:**      $$sgn\left( p \right): = \left\{ {\begin{array}{*{20}{l}} { - 1} \hfill & {{{{{{\mathrm{if}}}}}}\;{{{{{\mathrm{ES}}}}}}\left( p \right) \, < \, 0,} \hfill \\ 0 \hfill & {{{{{{\mathrm{if}}}}}}\;{{{{{\mathrm{ES}}}}}}\left( p \right) = 0,} \hfill \\ 1 \hfill & {{{{{{\mathrm{if}}}}}}\;{{{{{\mathrm{ES}}}}}}\left( p \right) \, > \, 0.} \hfill \end{array}} \right.$$

 **6:**      **if** $$ES \, \ne \, 0$$ **then**

 **7:**         **if** $$\mu _{H - D}\left( p \right) \in \left( {Q_3, + \infty } \right)$$ **then**

 **8:**          $${{{{{\mathrm{CS}}}}}}\left( {p,d} \right) = \left| {{{{{{\mathrm{PAS}}}}}}\left( {p,d} \right)} \right| \ast \left( {w_1 \ast sgn\left( p \right)} \right)$$

 **9:**         **else if** $\mu _{H - D}\left( p \right) \in |Q_2,Q_3|$ **then**

 **10:**          $${{{{{\mathrm{CS}}}}}}\left( {p,d} \right) = |{{{{{\mathrm{PAS}}}}}}\left( {p,d} \right)| \ast \left( {w_2 \ast sgn\left( p \right)} \right)$$

 **11:**         **else**

 **12:**          $${{{{{\mathrm{CS}}}}}}\left( {p,d} \right) = |{{{{{\mathrm{PAS}}}}}}\left( {p,d} \right)| \ast \left( {w_3 \ast sgn\left( p \right)} \right)$$

 **13:**      ** else**

 **14:**         $${{{{{\mathrm{CS}}}}}}\left( {p,d} \right) = {{{{{\mathrm{PAS}}}}}}\left( {p,d} \right)$$

    $\Rightarrow \, {\mathrm{CS}}$, Matrix consisting of calibrated pathway scores after drug treatment

 **15:** **return** $${{{{{\mathrm{CS}}}}}}$$

### Drug response prediction and prioritization

The methodology then aims at identifying drug candidates based on the predicted response of a patient to the simulated drug treatment. To do so, we input the modified features generated by the scoring algorithm in the trained ML model and re-evaluate the new class assignment of the patient.

Since the ML model has learnt to accurately differentiate between normal and disease samples, we expect that if a drug fails to affect a set of relevant pathways, the labels of the disease samples would remain unchanged. However, if the drug were to target a set of pathways dysregulated in a disease, we expect that the scoring algorithm could modify the scores so that they resemble those observed in control samples. Thus, by inputting these modified scores into the trained ML model, we can assess whether disease samples can now be classified as normal. Finally, after re-evaluating the predictions made by the ML model, we can rank promising drugs by the proportion of disease samples that are classified as normal as a proxy of the effectiveness of the drug.

### Validation and robustness analysis

Here, we outline the robustness experiments conducted to assess the ability of our methodology to identify drugs which are already FDA-approved or have been tested in clinical trials for each of the four cancer types (i.e., TCGA datasets).

First, to simulate drug treatment using the scoring algorithm described in Box 1, we used two different drug-target datasets: DrugBank (version 5.1.6)^49^ and DrugCentral (version 9.18.2020).^50^ For each of the datasets, we mapped drugs to DrugBank identifiers and protein targets to HGNC symbols. In total, we retrieved 1346 unique drugs and 4673 drug-target interactions from DrugBank and 638 unique drugs and 1481 drug-target interactions from DrugCentral. Here, we would like to note that both datasets are largely overlapping (Supplementary Fig. 11). We then used these drug-target interactions as the input to our methodology to simulate patient treatments (Fig. 1e).

For validation purposes, we used two ground-truth lists containing drug-disease pairs as true positives to verify the predictions made by our methodology (Fig. 1f). The first ground-truth list contained FDA-approved drugs for the four cancer types manually retrieved from the National Cancer Institute (https://www.cancer.gov/about-cancer/treatment/drugs/cancer-type) which we mapped to the two drug-target datasets previously described. The second ground-truth list contained drugs investigated in clinical trials for the four cancer datasets retrieved from the ClinicalTrials.gov website (downloaded on 16.04.2020). Table 3 lists the number of approved and clinically tested drugs present in both drug-target datasets across the four investigated cancers.

**Table 3:** Number of FDA-approved and clinically tested drugs present in both drug-target datasets across the four investigated cancers.

Dataset	DrugBank Approved	DrugBank Clinical trials	DrugCentral Approved	DrugCentral Clinical trials
BRCA	26/1346 (1.93%)	182/1346 (13.52%)	14/638 (2.19%)	115/638 (18.02%)
LIHC	5/1346 (0.37%)	50/1346 (3.71%)	1/638 (0.16%)	35/638 (5.49%)
PRAD	13/1346 (0.97%)	134/1346 (9.96%)	7/638 (1.10%)	84/638 (13.17%)
KIRC	8/1346 (0.60%)	44/1346 (3.26%)	3/638 (0.47%)	25/638 (3.91%)

The percentage for the number of FDA-approved/clinically investigated drugs for each cancer type over the total number of drugs present in the drug-target dataset is reported between parentheses.

As validation, both ground-truth lists were compared against the list of prioritized drugs that, according to our methodology, changed the predictions of 80% of the patients and subsequently classified them as normal. This threshold was selected as there were no drugs that changed the prediction for 90% or more of the patients with the parameters used by our scoring algorithm (Supplementary Figs. 7, 8). In addition, we would like to note that the vast majority of the drugs do not change the predictions for most patients. Thus, we were exclusively interested in assessing the ability of our approach to recover true positives (i.e., positive predictive value) from the list of prioritized drugs. However, since our methodology aims to prioritize drug candidates, it suffers from an early retrieval problem.^51^ Furthermore, only a small minority of drugs from the drug-target datasets can be used as positive labels for each of the indications, while the majority of drugs are not known to have therapeutic benefits for them, thus, creating a large imbalance between positive and negative labels. Due to these reasons, we maintain that the evaluation strategy we present is more suitable than other conventional metrics such as the receiver operating characteristic (ROC) curves.

To identify a set of weights for the three quartiles (i.e., *Q*_*1*_*, Q*_*2*_ *and Q*_*3*_ (see Box 1)) that perform well in three cancer test datasets, we followed a similar strategy to^26^ where we tested different weight combinations with the intention of assigning larger weights to pathways with significantly higher dysregulations between disease and normal samples. We would like to note that the purpose of using weights in the algorithm was to modify the pathway activity scores of the few but relevant pathways targeted by the drug while maintaining the underlying distribution of pathway scores (Supplementary Fig. 9). We performed the drug simulation and conducted this parameter optimization independently on the three cancer test datasets on DrugBank, the first of two drug-target datasets. Consequently, we found a set of weights (i.e., *W*_*1*_ = 20, *W*_*2*_ = 5, and *W*_*3*_ = 10 for *Q*_*3*_ (the upper quartile representing the most dysregulated pathways), *Q*_*2*_ (middle quartile), and *Q*_*1*_ (lower quartile), respectively), that yielded both a large proportion of true positives among the prioritized drugs and also performed better than any of the six methods we compared our methodology against, as described below. Finally, we validated whether this same set of weights could also yield a large proportion of true positives on the second drug-target dataset (i.e., DrugCentral) as well as the fourth cancer dataset (i.e, KIRC).

To test the robustness of our methodology, we replicated our experiments by generating one hundred sets of 1346 drugs (the size of the DrugBank dataset) where each drug was assigned to a randomly selected protein target (from the set of all HGNC symbols) with a random causal effect following the same distribution as the original dataset (i.e., activation or inhibition). Next, we compared the number of drugs prioritized by these permutation experiments against the number of drugs prioritized by our methodology for the DrugBank dataset in the three cancer test datasets. Since we use a method to generate pathway activity scores that ignores network topology (i.e., ssGSEA), we did not conduct a robustness analysis that focused on perturbing pathway networks.

### Performance comparison against equivalent drug-repurposing approaches

To evaluate our methodology, we compared it to six similar approaches that also employ transcriptomics data and pathway information to repurpose drugs on the BRCA and PRAD datasets^25,26^ (note that the LIHC dataset is not included in their analyses). In the first of the two studies,^25^ evaluated the ability of their methodology and four additional approaches to predict known drugs (i.e., FDA-approved or in advanced clinical trials) for breast and prostate cancer. Similarly,^26^ reported the ability of their approach to identify FDA-approved drugs on the same datasets. We were thus able to directly compare the proportion of true positives that were recovered by other approaches as reported in the aforementioned studies against the proportion recovered by our approach.

### Implementation

We performed ssGSEA with the Python package, GSEApy (version 0.9.12; https://github.com/zqfang/gseapy) and generated the ML models using scikit-learn.^52^ We would like to note that ssGSEA does not take the topology of the pathways into account.

## Supplementary information

Supplementary Information

Supplementary Data 1

## Data Availability

Data used in this manuscript are available at https://github.com/sepehrgolriz/simdrugs under the Apache 2.0 License.

## Code availability

Source code used in this manuscript is available at https://github.com/sepehrgolriz/simdrugs under the Apache 2.0 License.
