# Supplementary material for: Using predictive machine learning models for drug response simulation by calibrating patient-specific pathway signatures
Source: NPJ Syst Biol Appl. 2021 Oct 27;7:40. doi: 10.1038/s41540-021-00199-1 (PMC8551267; doi:10.1038/s41540-021-00199-1)
Supplement: Supplementary file 1 — Supplementary Information [file 41540_2021_199_MOESM1_ESM.pdf]

Supplementary File

Supplementary Tables

Dr. Insight Performance		
**Dataset**	**TCGA BRCA**	**TCGA PRAD**
**# of proposed drug treatments**	70	69
**# identified ground-truth drug treatments**	15	11
**Proportion of true positives (%)**	**21.42**	**15.94**

Supplementary Table 1. Number of approved drugs or drugs in clinical trials (i.e., ground-truth drug treatments) recovered using Dr. Insight on the BRCA and PRAD datasets. See the **Supplementary Text** for a detailed description of the information reported in each of the rows. The results of this table are reported in Chan *et al*. (2019) (Table S5.11) https://doi.org/10.1093/bioinformatics/btz006.

CMap performance on BRCA								
**Gene signature sizes (threshold)**	50	100	200	300	400	600	800	1000
**# of proposed drug treatments**	56	52	55	67	74	74	71	56
**# identified ground-truth drug treatments**	5	6	3	6	5	6	6	5
**Proportion of true positives (%)**	8.92	**11.53**	5.45	8.92	6.75	8.1	8.45	8.92

Supplementary Table 2. Number of approved drugs or drugs in clinical trials (i.e., ground-truth drug treatments) recovered using CMap on the BRCA dataset. The proportion of true positives highlighted in bold indicates the highest percentage amongst all parameters (i.e., threshold gene set size). See the **Supplementary Text** for a detailed description of the information reported in each of the rows. The results of this table are reported by Chan *et al*. (2019) (Table S5.1) https://doi.org/10.1093/bioinformatics/btz006.

CMap performance on PRAD								
**Gene signature sizes (threshold)**	50	100	200	300	400	600	800	1000
**# of proposed drug treatments**	52	83	65	66	80	73	76	71
**# identified ground-truth drug treatments**	8	11	7	7	9	10	8	9
**Proportion of true positives (%)**	**15.38**	13.25	10.76	10.60	11.25	13.69	10.52	12.67

Supplementary Table 3. Number of approved drugs or drugs in clinical trials (i.e., ground-truth drug treatments) recovered using CMap on the PRAD dataset. The proportion of true positives highlighted in bold indicates the highest percentage amongst all parameters (i.e., threshold gene set size). See the **Supplementary Text** for a detailed description of the information reported in each of the rows. The results of this table are reported by Chan *et al*. (2019) (Table S5.2) https://doi.org/10.1093/bioinformatics/btz006.

sscMap performance on BRCA								
**Gene signature sizes (threshold)**	50	100	200	300	400	600	800	1000
**# of proposed drug treatments**	6	19	659	998	1185	1316	1404	1436
**# identified ground-truth drug treatments**	0	1	31	45	61	63	72	77
**Proportion of true positives (%)**	0	5.26	4.70	4.50	5.14	4.78	5.12	**5.36**

Supplementary Table 4. Number of approved drugs or drugs in clinical trials (i.e., ground-truth drug treatments) recovered using sscMap on the BRCA dataset. The proportion of true positives highlighted in bold indicates the highest percentage amongst all parameters (i.e., threshold gene set size). See the **Supplementary Text** for a detailed description of the information reported in each of the rows. The results of this table are reported by Chan *et al*. (2019) (Table S5.4) https://doi.org/10.1093/bioinformatics/btz006.

sscMap performance on PRAD								
**Gene signature sizes (threshold)**	50	100	200	300	400	600	800	1000
**# of proposed drug treatments**	8	18	100	177	202	381	653	810
**# identified ground-truth drug treatments**	1	4	9	11	14	17	21	25
**Proportion of true positives (%)**	12.5	**22.22**	9	6.21	6.93	4.46	3.21	3.08

Supplementary Table 5. Number of approved drugs or drugs in clinical trials (i.e., ground-truth drug treatments) recovered using sscMap on the PRAD dataset. The proportion of true positives highlighted in bold indicates the highest percentage amongst all parameters (i.e., threshold gene set size). See the **Supplementary Text** for a detailed description of the information reported in each of the rows. The results of this table are reported by Chan *et al*. (2019) (Table S5.5) https://doi.org/10.1093/bioinformatics/btz006.

NFFinder performance on BRCA								
**Gene signature sizes (threshold)**	50	100	200	300	400	600	800	1000
**# of proposed drug treatments**	329	285	623	782	651	854	1007	1069
**# identified ground-truth drug treatments**	26	24	40	45	43	54	66	62
**Proportion of true positives (%)**	7.90	**8.42**	6.42	5.75	6.60	6.32	6.65	5.59

Supplementary Table 6. Number of approved drugs or drugs in clinical trials (i.e., ground-truth drug treatments) recovered using NFFinder on the BRCA dataset. The proportion of true positives highlighted in bold indicates the highest percentage amongst all parameters (i.e., threshold gene set size). See the **Supplementary Text** for a detailed description of the information reported in each of the rows. The results of this table are reported by Chan *et al*. (2019) (Table S5.7) https://doi.org/10.1093/bioinformatics/btz006.

NFFinder performance on PRAD								
**Gene signature sizes (threshold)**	50	100	200	300	400	600	800	1000
**# of proposed drug treatments**	347	592	548	717	529	719	842	783
**# identified ground-truth drug treatments**	18	28	32	34	32	38	42	38
**Proportion of true positives (%)**	5.18	4.72	**5.83**	4.50	4.74	5.28	4.98	4.85

Supplementary Table 7. Number of approved drugs or drugs in clinical trials (i.e., ground-truth drug treatments) recovered using NFFinder on the PRAD dataset. The proportion of true positives highlighted in bold indicates the highest percentage amongst all parameters (i.e., threshold gene set size). See the **Supplementary Text** for a detailed description of the information reported in each of the rows. The results of this table are reported by Chan *et al*. (2019) (Table S5.8) https://doi.org/10.1093/bioinformatics/btz006.

Cogena		
**Cogena**	TCGA BRCA	TCGA PRAD
**# of proposed drug treatments**	335	982
**# identified ground-truth drug treatments**	30	5
**Proportion of true positives (%)**	**8.95**	**0.50**

Supplementary Table 8. Number of approved drugs or drugs in clinical trials (i.e., ground-truth drug treatments) recovered using Cogena on the BRCA and PRAD datasets. See the **Supplementary Text** for a detailed description of the information reported in each of the rows. The results of this table are reported by Chan *et al*. (2019) (Table S5.10) https://doi.org/10.1093/bioinformatics/btz006.

Chen *et al.* (2016) study performance			
**Dataset**	**Prioritized**	**Approved (total)**	**Proportion of true positives (%)**
**BRCA**	2435	20 (20)	20/2435 **(0.81%)**
**PRAD**	2500	10 (11)	10/2500 **(0.40%)**

Supplementary Table 9. Number of approved drugs recovered reported by Chen *et al.* (2016) on the BRCA and PRAD datasets. The results from this table are reported in Table 1 of the paper (https://doi.org/10.1186/s12920-016-0212-7).

Method	BRCA	PRAD
**Dr. Insight**	21.42 (%)	15.94 (%)
**CMap**	6.75 - 11.53 (%)	10.52 - 15.38 (%)
**sscMap**	0 - 5.36 (%)	3.08 - 22.22 (%)
**NFFinder**	5.59 - 8.42 (%)	4.5 - 5.83 (%)
**Cogena**	8.95 (%)	0.50 (%)
**Chen *et al.* (2016)**	0.81 (%)	0.40 (%)

Supplementary Table 10. Summary of performance for all methods benchmarked on the BRCA and PRAD datasets. Performances are measured as % of approved drugs or drugs in clinical trials recovered and are taken from Supplementary Tables 1-9.

DrugCentral (638 drugs)						
**TCGA dataset**	**Replaced weight**	**Weight sets (W1_W2_W3)**	**# Prioritized**	**# Clinical trials (total)**	**# Approved (total)**	**Proportion of true positives (%)**
**BRCA**	**W1**	1_5_10	0	-	-	-
		5_5_10	0	-	-	-
		10_5_10	3	1(115)	-	1/3(33%)
		15_5_10	7	2(115)	-	2/7(28%)
	**W2**	20_1_10	17	4(115)	1(14)	5/17(29%)
		20_10_10	29	5(115)	1(14)	6/29(20%)
		20_15_10	31	5(115)	1(14)	6/31(19%)
		20_20_10	46	6(115)	1(14)	7/46(15%)
	**W3**	20_5_1	16	4(115)	1(14)	5/16(31%)
		20_5_5	17	4(115)	1(14)	5/17(29%)
		20_5_15	18	4(115)	1(14)	5/18(27%)
		20_5_20	19	4(115)	1(14)	5/18(27%)
**LIHC**	**W1**	1_5_10	14	1(35)	1(1)	2/14(14%)
		5_5_10	16	1(35)	1(1)	2/16(12%)
		10_5_10	19	2(35)	1(1)	3/19(15%)
		15_5_10	19	2(35)	1(1)	3/19(15%)
	**W2**	20_1_10	19	2(35)	1(1)	3/19(15%)
		20_10_10	20	2(35)	1(1)	3/20(15%)

		20_15_10	21	2(35)	1(1)	3/21(14%)
		20_20_10	22	2(35)	1(1)	3/22(13%)
	W3	20_5_1	9	1(35)	-	1/9(11%)
		20_5_5	10	1(35)	1(1)	2/10(20%)
		20_5_15	20	2(35)	1(1)	3/20(15%)
		20_5_20	20	2(35)	1(1)	3/20(15%)
PRAD	W1	1_5_10	1	1(84)	-	1/1(100%)
		5_5_10	1	1(84)	-	1/1(100%)
		10_5_10	1	1(84)	-	1/1(100%)
		15_5_10	9	3(84)	-	3/9(33%)
	W2	20_1_10	11	2(84)	-	2/11(18%)
		20_10_10	19	3(84)	1(7)	4/19(21%)
		20_15_10	40	7(84)	1(7)	8/40(20%)
		20_20_10	41	8(84)	1(7)	9/41(21%)
	W3	20_5_1	16	3(84)	-	3/16(18%)
		20_5_5	16	3(84)	-	3/16(18%)
		20_5_15	32	7(84)	1(7)	8/32(25%)
		20_5_20	32	7(84)	1(7)	8/32(25%)

Supplementary Table 11. Number of FDA-approved and clinically tested drugs recovered across the three investigated cancers using different weights in the DrugCentral dataset. In the fourth column (i.e., # Prioritized), we report the number of drugs that changed the predictions for at least 80% of the patients for each cancer type.

DrugBank (1346 drugs)						
TCGA dataset	Replaced weight	Weight sets (W1_W2_W3)	# Prioritized	# Clinical trials (total)	# Approved (total)	Proportion of true positives (%)
BRCA	W1	1_5_10	22	4(182)	0(26)	4/22(18%)
		5_5_10	55	8(182)	2(26)	10/55(18%)
		10_5_10	81	12(182)	2(26)	14/81(17%)
		15_5_10	92	14(182)	2(26)	16/92(17%)
	W2	20_1_10	124	21(182)	3(26)	24/124(19%)
		20_10_10	142	24(182)	3(26)	27/142(19%)
		20_15_10	148	24(182)	3(26)	27/148(18%)
		20_20_10	172	27(182)	3(26)	30/172(14%)
	W3	20_5_1	85	16(182)	3(26)	19/85(22%)
		20_5_5	89	18(182)	3(26)	21/89(23%)
		20_5_15	142	25(182)	3(26)	28/142(19%)
		20_5_20	143	25(182)	3(26)	28/142(19%)
LIHC	W1	1_5_10	67	9(50)	2(5)	11/67(16%)
		5_5_10	70	10(50)	2(5)	12/70(17%)
		10_5_10	70	9(50)	2(5)	11/70(16%)
		15_5_10	73	10(50)	2(5)	12/73(16%)
	W2	20_1_10	71	11(50)	2(5)	13/71(18%)
		20_10_10	81	13(50)	2(5)	15/81(18%)
		20_15_10	89	14(50)	2(5)	16/89(18%)
		20_20_10	93	14(50)	2(5)	16/93(18%)
	W3	20_5_1	36	8(50)	1(5)	9/36(25%)
		20_5_5	46	8(50)	2(5)	10/46(21%)
		20_5_15	101	13(50)	3(5)	16/101(19%)
		20_5_20	117	13(50)	3(5)	16/101(19%)
PRAD	W1	1_5_10	3	-	-	-
		5_5_10	7	-	-	-
		10_5_10	19	1(134)	-	1/19(5%)

		15_5_10	27	2(134)	-	2/27(7%)
	W2	20_1_10	46	10(134)	-	10/46(22%)
		20_10_10	82	19(134)	2(13)	21/82(25%)
		20_15_10	59	8(134)	2(13)	10/59(16%)
		20_20_10	94	19(134)	2(13)	21/94(22%)
	W3	20_5_1	66	16(134)	-	16/66(24%)
		20_5_5	64	15(134)	-	15/64(23%)
		20_5_15	59	8(134)	2(13)	10/59(17%)
		20_5_20	63	8(134)	0(13)	8/59(14%)

Supplementary Table 12. Number of FDA-approved and clinically tested drugs recovered across the three investigated cancers using different weights in the DrugBank dataset. In the fourth column (i.e., # Prioritized), we report the number of drugs that changed the predictions for at least 80% of the patients for each cancer type.

	**Weights 10(Q3), 5(Q2), 2(Q1)**							
**-**	**DrugBank**				**DrugCentral**			
**Dataset**	**Prioritized**	**Approved (total)**	**Clinical trials (total)**	**Proportion of true positives (%)**	**Prioritized**	**Approved (total)**	**Clinical trials (total)**	**Proportion of true positives (%)**
**BRCA**	20	3(26)	2(182)	5/20**(25%)**	2	0(14)	1(115)	1/2**(50%)**
**LIHC**	27	1(5)	6(50)	7/27**(25.95%)**	4	0(1)	1(35)	1/4**(25%)**
**PRAD**	17	0(13)	0(134)	0/17**(0%)**	0	0(7)	0(84)	**0**

Supplementary Table 13. Number of FDA-approved and clinically tested drugs recovered for both drug-target datasets across the three investigated cancers. In the columns labelled "Prioritized", we report the number of drugs that changed the predictions for at least 80% of the patients for each cancer type. A different set of weights were used than the ones used to generate the results of Table 2, leading to comparatively better results for the BRCA and LIHC datasets, but resulting in no true positives recovered for PRAD.

**-**	**Weights 1(Q3), 1(Q2), 1(Q1)**							
**-**	**DrugBank**				**DrugCentral**			
**Dataset**	**Prioritized**	**Approved (total)**	**Clinical trials (total**	**Proportion of true positives (%)**	**Prioritized**	**Approved (total)**	**Clinical trials (total**	**Proportion of true positives (%)**
**BRCA**	0	0(26)	0(182)	**0**	0	0(14)	0(115)	**0**
**LIHC**	0	0(5)	0(50)	**0**	0	0(1)	0(35)	**0**
**PRAD**	0	0(13)	0(134)	**0**	0	0(7)	0(84)	**0**

Supplementary Table 14. Number of FDA-approved and clinically tested drugs recovered for both drug-target datasets across the three investigated cancers. In the columns labelled "Prioritized", we report the number of drugs that changed the predictions for at least 80% of the patients for each cancer type. Here, we set all weights equal to one and find that there are no prioritized drugs for any of the three cancer datasets.

**Pathway**	**Target**	**Pathway- level drug effect**	**Pathway activity in patients relative to controls**
Gap junction	PDGFRB, RAF1	Inhibition	Upregulated
Fc gamma R mediated phagocytosis	RAF1	Inhibition	Upregulated
Phospholipase D signaling pathway	INSR, PDGFRB, KIT, RAF1	Inhibition	Upregulated
Thyroid hormone signaling pathway	RAF1	Inhibition	Upregulated
Thyroid cancer	BRAF, RET	Inhibition	Upregulated

Hepatitis B	BRAF, RAF1	Inhibition	Upregulated
Human papillomavirus infection	PDGFRB, RAF1	Inhibition	Upregulated
Focal adhesion	BRAF, FLT4, KDR, PDGFRB, RAF1, FLT1	Inhibition	Upregulated
Renal cell carcinoma	BRAF, RAF1	Inhibition	Upregulated
Glioma	BRAF, PDGFRB, RAF1	Inhibition	Upregulated
Axon guidance	RAF1	Inhibition	Upregulated
Endocrine resistance	BRAF, RAF1	Inhibition	Upregulated
Breast cancer	BRAF, FLT4, KIT, RAF1	Inhibition	Upregulated
Sphingolipid signaling pathway	RAF1	Inhibition	Upregulated
Autophagy animal	RAF1	Inhibition	Upregulated
mTOR signaling pathway	INSR, BRAF, RAF1	Inhibition	Upregulated
GnRH signaling pathway	RAF1	Inhibition	Upregulated
Pathways in cancer	FLT3, BRAF, FLT4, PDGFRB, KIT, RAF1, RET	Inhibition	Upregulated
Chronic myeloid leukemia	BRAF, RAF1	Inhibition	Upregulated
Choline metabolism in cancer	PDGFRB, RAF1	Inhibition	Upregulated
Bladder cancer	BRAF, RAF1	Inhibition	Upregulated
Non small cell lung cancer	BRAF, RAF1	Inhibition	Upregulated
Gastric cancer	BRAF, RAF1	Inhibition	Upregulated
Cushing syndrome	BRAF	Inhibition	Upregulated
VEGF signaling pathway	KDR, RAF1	Inhibition	Upregulated
Hepatocellular carcinoma	BRAF, RAF1	Inhibition	Upregulated
MAPK signaling pathway	INSR,FLT3,BRAF,FLT4,KDR,PDGFRB,KIT,RAF1,	Inhibition	Upregulated
Regulation of actin cytoskeleton	BRAF, PDGFRB, RAF1	Inhibition	Upregulated
Human immunodeficiency virus 1 infection	RAF1	Inhibition	Upregulated
Relaxin signaling pathway	RAF1	Inhibition	Upregulated
Estrogen signaling pathway	RAF1	Inhibition	Upregulated
Progesterone mediated oocyte maturation	BRAF, RAF1	Inhibition	Upregulated
MicroRNAs in cancer	PDGFRB, RAF1	Inhibition	Upregulated
Neurotrophin signaling pathway	BRAF, RAF1	Inhibition	Upregulated
Alcoholism	BRAF, RAF1	Inhibition	Upregulated
Fc epsilon RI signaling pathway	RAF1	Inhibition	Upregulated
Apoptosis	RAF1	Inhibition	Upregulated
Cellular senescence	RAF1	Inhibition	Upregulated
Colorectal cancer	BRAF, RAF1	Inhibition	Upregulated
Long term depression	BRAF, RAF1	Inhibition	Upregulated
Melanogenesis	KIT, RAF1	Inhibition	Upregulated

Supplementary Table 15. Effect of Sorafenib on pathway targets in the LIHC dataset. The first column corresponds to the pathways that contain protein targets of Sorafenib while the second column corresponds to the specific protein targets of the drug. The third column corresponds to the effect of the drug on the pathway based on its effect on the target. In this case, all pathways are inhibited as all protein

targets are inhibited by Sorafenib. Finally, the last column presents a relative comparison between the pathway activity observed in patients vs. controls: downregulated corresponds to lower pathway activity and upregulated corresponds to the opposite.

Dataset	Normal samples	Tumor samples	Reference	DOI
**BRCA**	113	1102	(The Cancer Genome Atlas Network, 2012)	https://doi.org/10.1038/nature11412
**LIHC**	50	371	(The Cancer Genome Atlas Research Network, 2017)	https://doi.org/10.1016/j.cell.2017.05.046
**PRAD**	52	498	(The Cancer Genome Atlas Research Network, 2015)	https://doi.org/10.1016/j.cell.2015.10.025
**KIRC**	72	538	(The Cancer Genome Atlas Research Network, 2013)	https://doi.org/10.1038/nature12222

Supplementary Table 16. Number of normal and tumor samples in the TCGA datasets used in this work.

Parameter	Configuration
Method	rank
Minimum size of gene set	15
Maximum size of gene set	3000

Supplementary Table 17. Parameter configuration settings for running ssGSEA with GSEApy (version 0.9.12).

Dr. Insight (Chan *et al.* (2019))				
**Dataset**	**Normal samples**	**Tumor samples**	**Reference**	**DOI**
**BRCA**	111	1099	(The Cancer Genome Atlas Network, 2012)	https://doi.org/10.1038/nature11412
**PRAD**	52	498	(The Cancer Genome Atlas Research Network, 2015)	https://doi.org/10.1016/j.cell.2015.10.025

Supplementary Table 18. Number of normal and tumor samples in the TCGA datasets used in the Chan *et al.* (2019) study. Datasets were retrieved through the Genomic Data Commons (GDC; https://gdc.cancer.gov) by Chan *et al.* (2019). Log transformed TCGA level-3 normalized count data was used in their study. Study details can be found at https://doi.org/10.1093/bioinformatics/btz006.

Chen *et al.* (2016)		
	**Breast Cancer**	**Prostate Cancer**
Total compounds	3678	4228
Compounds that are FDA-approved drugs	632	676
Compounds that are FDA-approved drugs for target disease	20	11
Compounds that are in clinical trial for target disease	154	106
Total number of pathways	287	

Supplementary Table 19. Information about the chemicals used by Chen *et al.* (2016). Details of the approach can be found at https://doi.org/10.1186/s12920-016-0212-7.

Dr. Insight (Chan *et al.* (2019))		
	**PRAD**	**BRCA**
FDA-approved drugs	7	9
Clinical trials drugs	47	63
Total number of drugs	54	72
Total number of pathways	222	

Supplementary Table 20. Information about the chemicals used by Chan *et al.* (2019) study. Study details can be found at https://doi.org/10.1093/bioinformatics/btz006.

Supplementary Text

1. Drug simulation scenario

Suppose you have a score of 0.2 for patient *A* on pathway *X*. If a drug is activating the pathway and the mean difference between healthy and disease groups is large, this pathway will be in the first quartile and the initial pathway score will be multiplied by a higher weight, (e.g., 3). Thus, the modified score for patient *A* on pathway *X* (originally 0.2) will be 0.6. However, if the mean difference between healthy and disease groups is not large, the weight will be smaller (e.g., 2) and the modified score for the patient on pathway *X* will be 0.4. These steps are repeated for all pathways which contain protein targets of a particular drug for a given patient. Finally, all modified scores are then passed to the classifier to determine whether the patient is subsequently classified as normal.

2. Measurements reported by equivalent approaches

Below, we describe each of the measurements reported by studies on similar drug-repurposing approaches that can be found in **Supplementary Tables 1-8**.

Gene signature sizes (threshold): This refers to the size of the list of query gene signatures used to evaluate the drug repurposing performance of CMap, sscMap and NFFinder. Specifically, the gene signatures were composed of the top- and bottom-ranked most differentially expressed genes of varying sizes. As the CMap, sscMap and NFFinder methods did not provide specific recommendations for the size of query gene signatures in their original work, the developers of the Dr. Insight method used gene lists of varying sizes (50, 100, 200, 300, 400, 600, 800 and 1000 Affymetrix probes) to evaluate the drug repurposing performance of CMap, sscMap and NFFinder and compare them with their method.

Number of identified drug treatments: This number refers to the drugs which were prioritized by different methods (i.e., NFFinder, CMap, sscMap, cogena, Dr. Insight).

Number of identified ground-truth drug treatments: This number refers to the number of FDA-approved drugs and clinical-trial drugs from the "# Identified drug treatments".

Proportion of true positives (%): This proportion refers to the “# identified ground-truth drug treatments” over the “# Identified drug treatments”.

Supplementary Figures

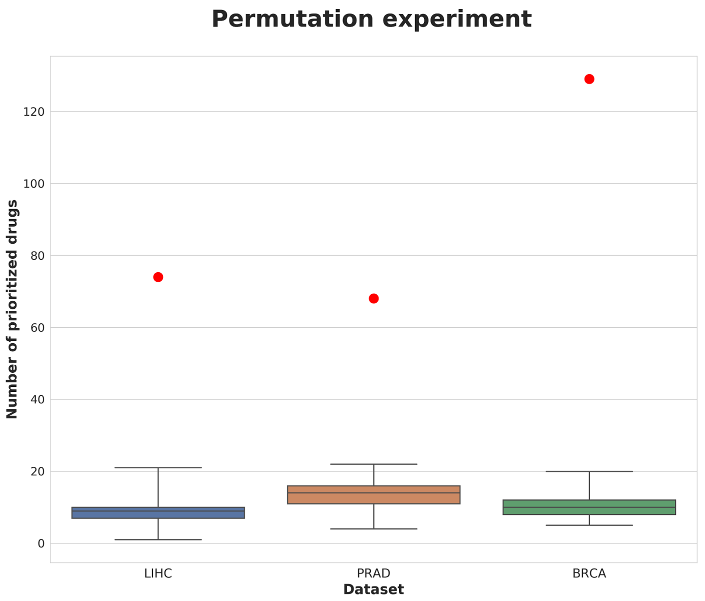

Supplementary Figure 1. Comparison of the results of the permutation experiments against the number of prioritized drugs in DrugBank for the three cancer test datasets. While the three boxplots correspond to the number of prioritized drugs in the 100 permutation experiments for each of the three datasets, the number of prioritized drugs in the original DrugBank dataset has been indicated with a red circle. The number of prioritized drugs from DrugBank is significantly higher than for any of the permutations experiments. *p*-values have been omitted as all permutation experiments yielded a lower number of prioritized drugs compared to the original dataset and thus, *p*-values would be dominated by the number of experiments (i.e., 100 experiments would yield a *p*-value of 0.01, and 1,000 experiments would yield a *p*-value of 0.001). We would like to note that we compare the permutation experiments against DrugBank as the number of simulated drugs is equal to the size of this dataset (1,346 drugs). Furthermore, a comparison to the DrugCentral dataset would yield an even greater difference in the number of prioritized drugs as DrugCentral is smaller in size (638 drugs).

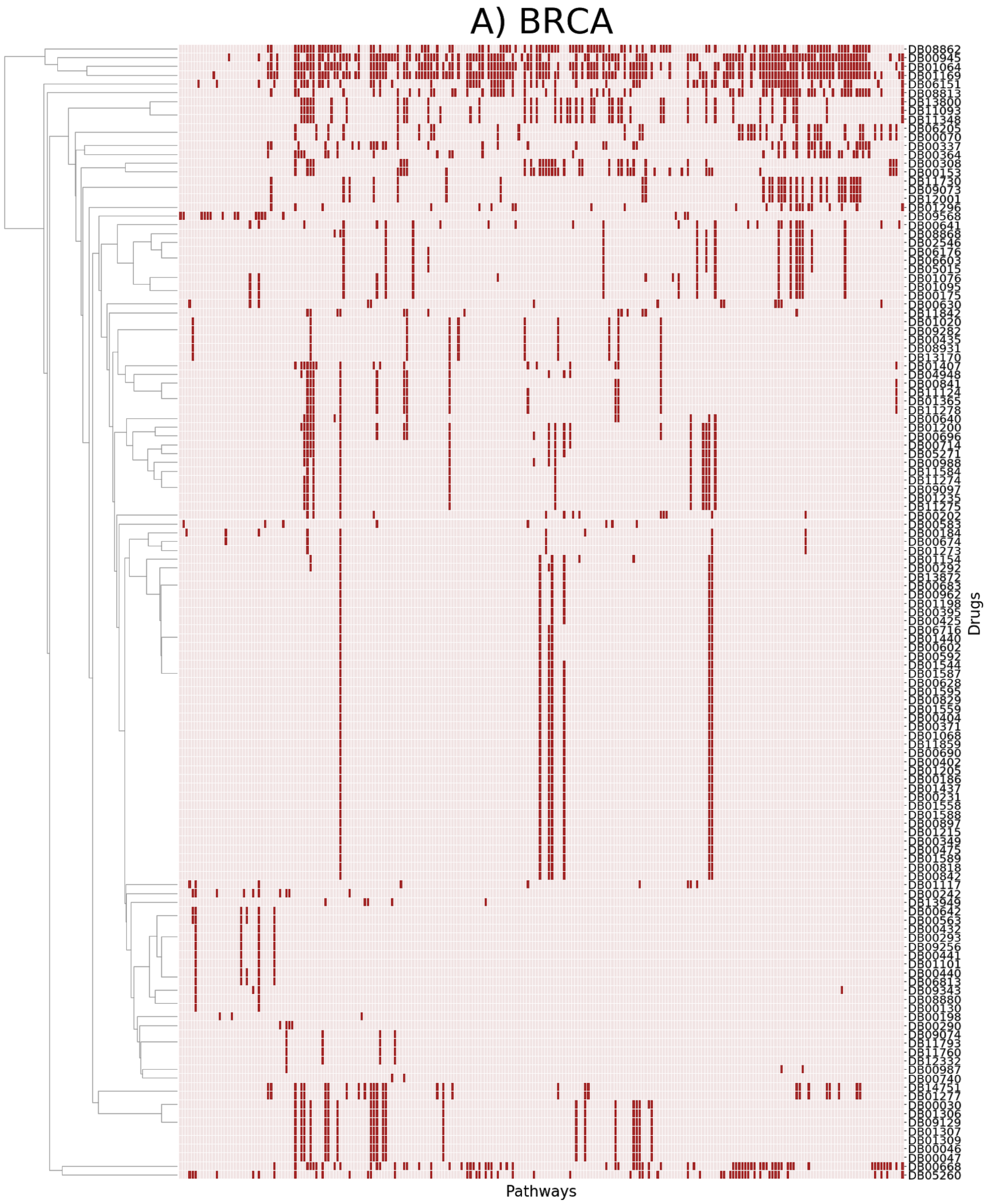

Supplementary Figure 2. Pathways targeted by the prioritized drugs in DrugBank for BRCA. The X-axis corresponds to the pathways targeted by any of the prioritized drugs (KEGG pathways not targeted by any prioritized drug have been omitted for better visualization). Drugs (Y-axis) have been clustered based on the pathways they target. Due to the large number of pathways, we have clustered pathway groups together for visualization purposes. Black cells correspond to pathways targeted for each drug. Details about each pathway are displayed in the following Jupyter notebook https://github.com/sepehrgolriz/simdrugs/blob/main/scripts_and_notebooks/heatmaps.ipynb.

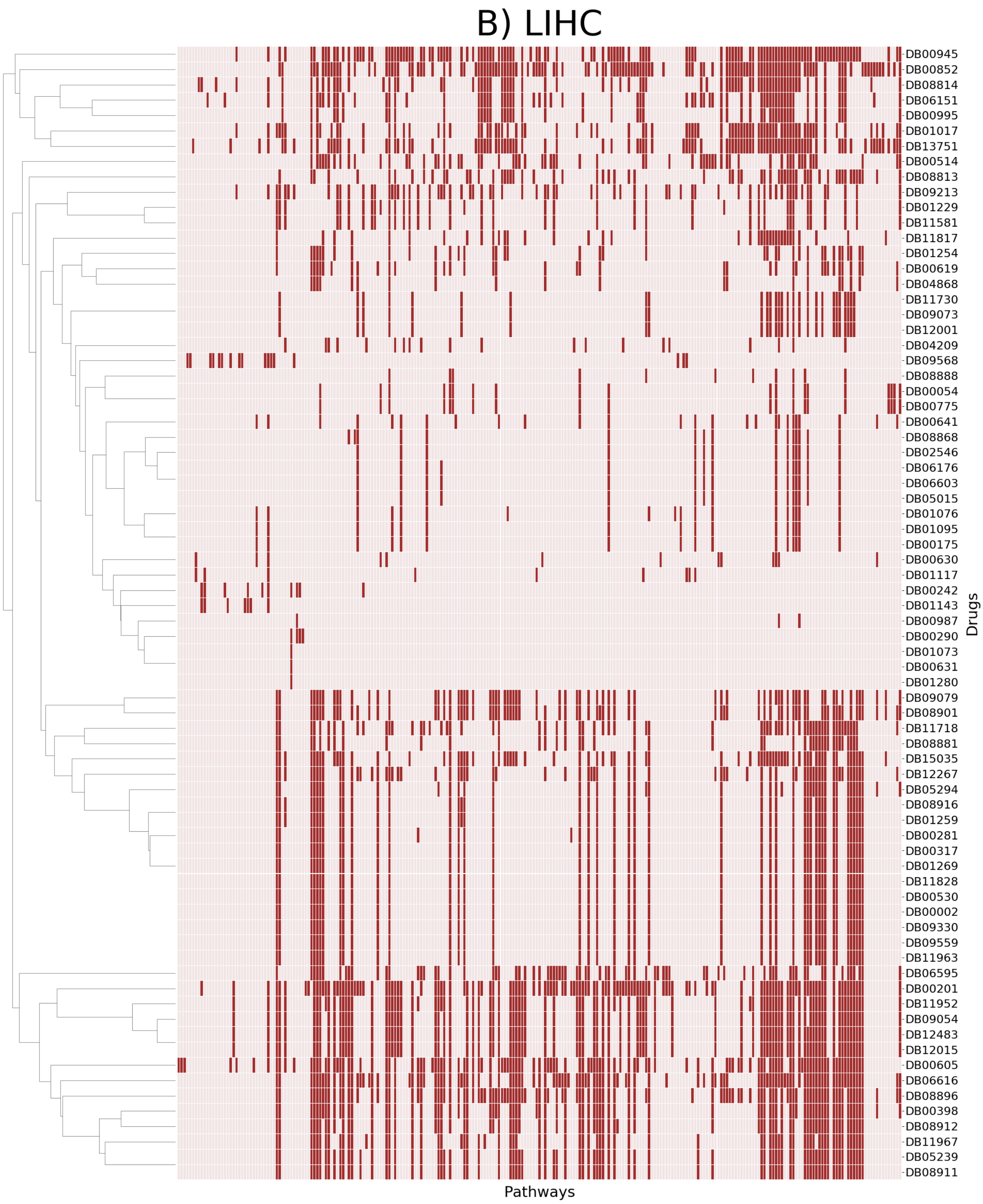

Supplementary Figure 3. Pathways targeted by the prioritized drugs in DrugBank for LIHC. The X-axis corresponds to the pathways targeted by any of the prioritized drugs (KEGG pathways not targeted by any prioritized drug have been omitted for better visualization). Drugs (Y-axis) have been clustered based on the pathways they target. Due to the large number of pathways, we have clustered pathway groups together for visualization purposes. Black cells correspond to pathways targeted for each drug. Details about each pathway are displayed in the following Jupyter notebook https://github.com/sepehrgolriz/simdrugs/blob/main/scripts_and_notebooks/heatmaps.ipynb.

C) PRAD

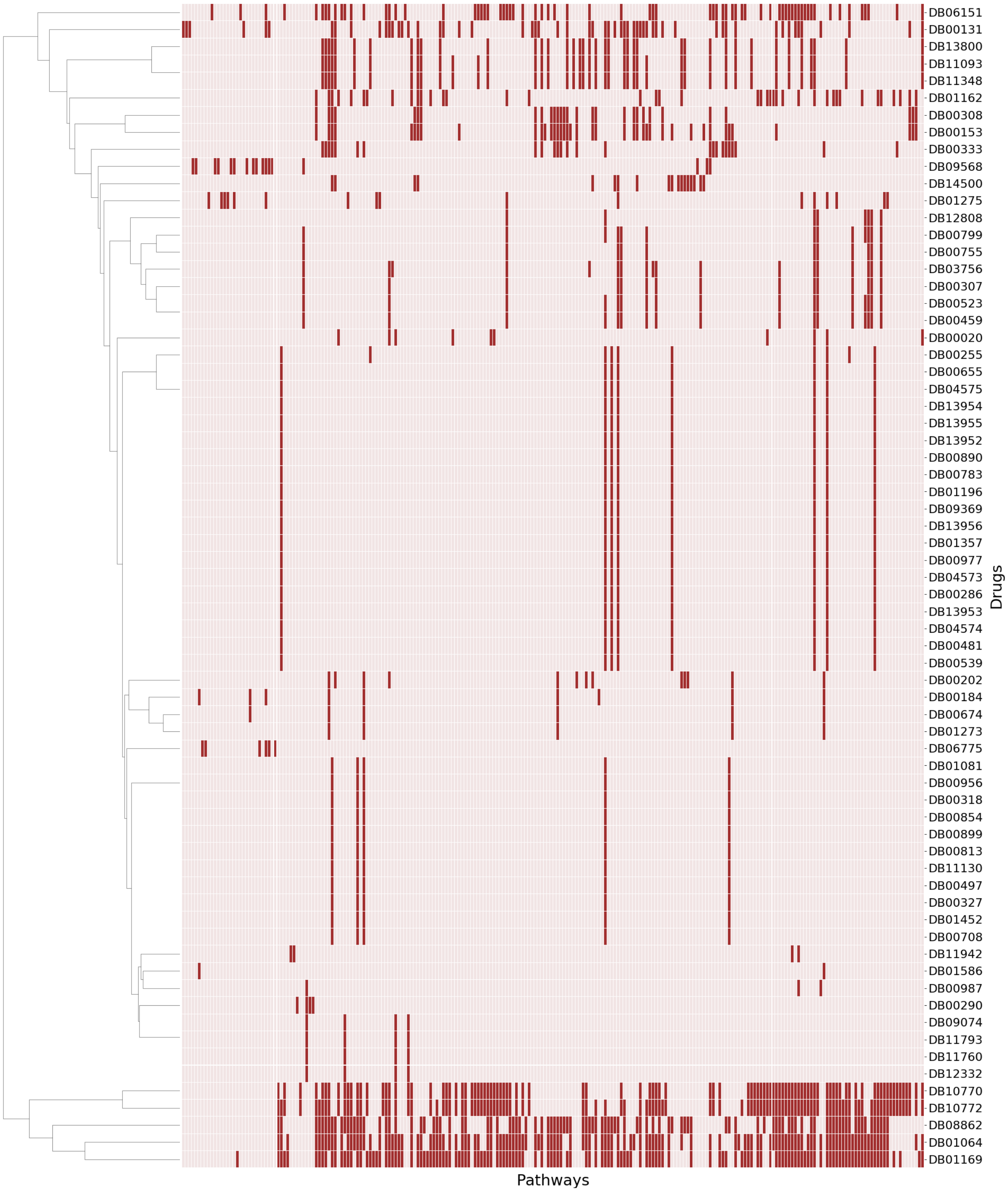

Supplementary Figure 4. Pathways targeted by the prioritized drugs in DrugBank for PRAD. The X-axis corresponds to the pathways targeted by any of the prioritized drugs (KEGG pathways not targeted by any prioritized drug have been omitted for better visualization). Drugs (Y-axis) have been clustered based on the pathways they target. Due to the large number of pathways, we have clustered pathway groups together for visualization purposes. Black cells correspond to pathways targeted for each drug. Details about each pathway are displayed in the following Jupyter notebook https://github.com/sepehrgolriz/simdrugs/blob/main/scripts_and_notebooks/heatmaps.ipynb.

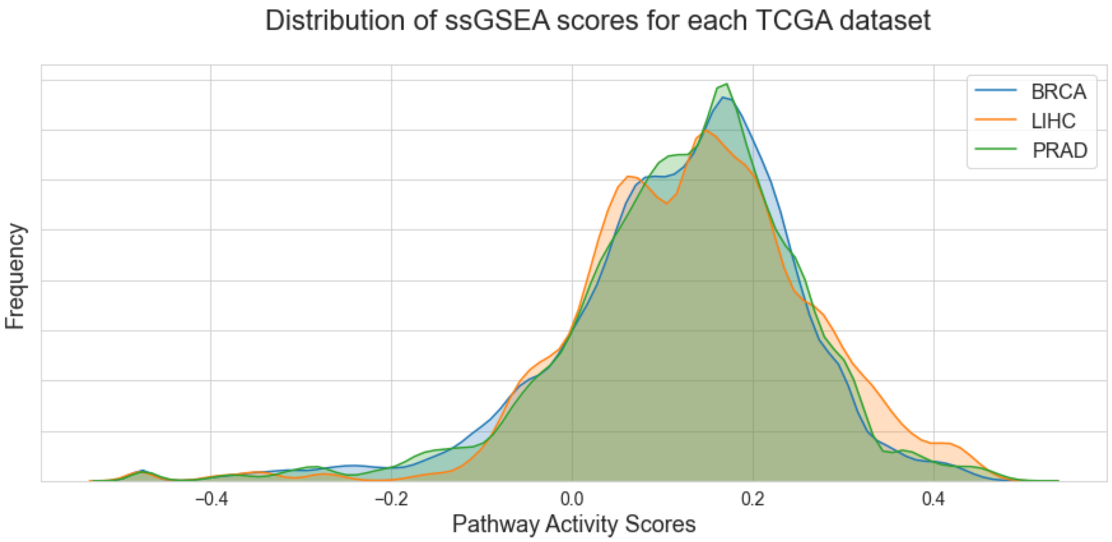

Supplementary Figure 5. Distribution of pathway activity scores for each of the three test datasets.

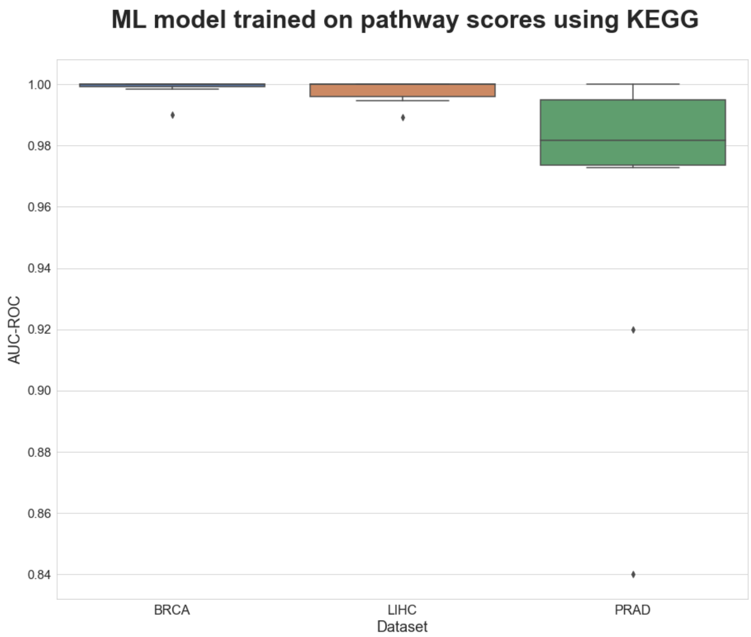

Supplementary Figure 6. Prediction performance measured as AUC-ROC values of an elastic net classifier (tumor vs. normal samples) trained on the three test TCGA datasets using pathway activity scores from ssGSEA run on KEGG. Each boxplot shows the distribution of the AUCs over 10 repeats of the 10-fold cross-validation procedure. The same classifiers yielded equivalent AUC-PR values (data not shown). Similar results were obtained in the KIRC dataset (see https://doi.org/10.3389/fgene.2019.0120 Figure 4).

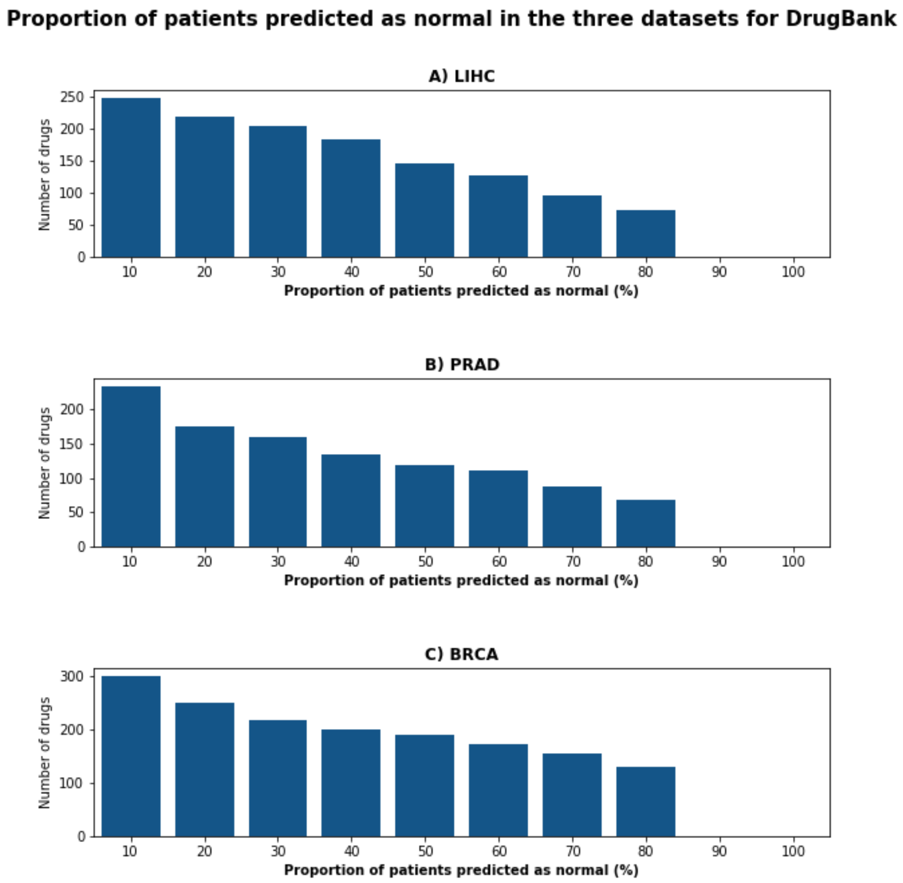

Supplementary Figure 7. Proportion of patients predicted as normal for each cancer test dataset using DrugBank. Only a fraction of all drugs in DrugBank changed the predictions of 10% of the patients to normal. As the proportion of the samples changed increases, the number of prioritized drugs decreases to 19 for all three datasets.

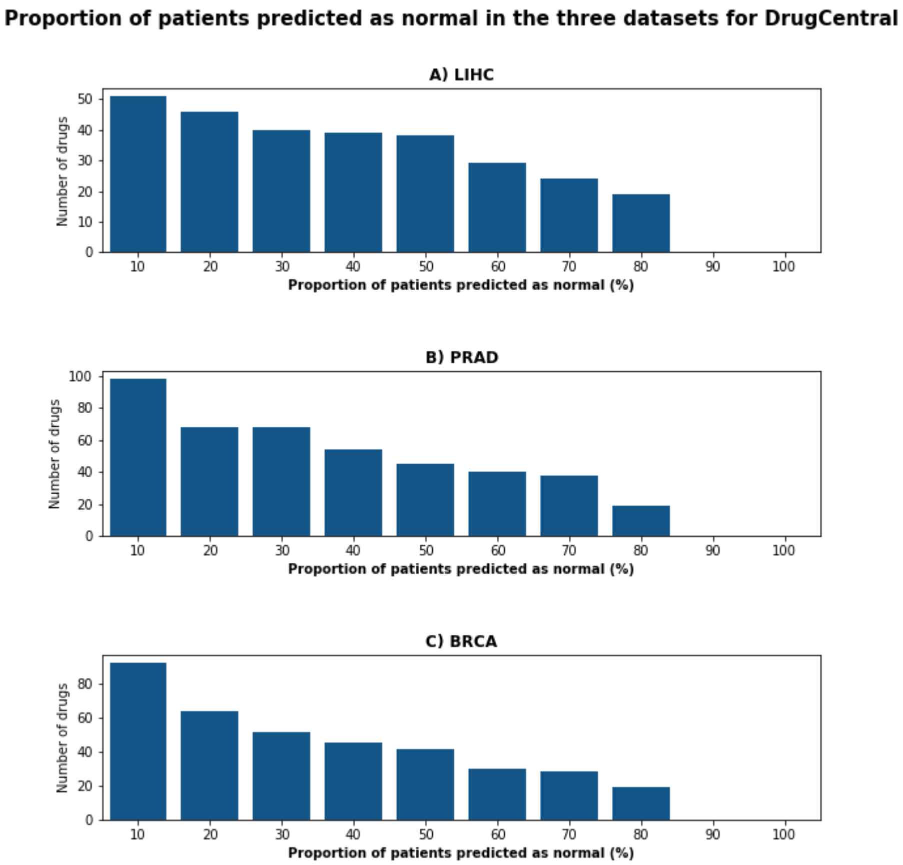

Supplementary Figure 8. Proportion of patients predicted as normal for each cancer test dataset using DrugCentral. Only a fraction of all drugs in DrugCentral changed the predictions of 10% of the patients to normal. As the proportion of the samples changed increases, the number of prioritized drugs decreases to a shortlist of drugs for all three datasets.

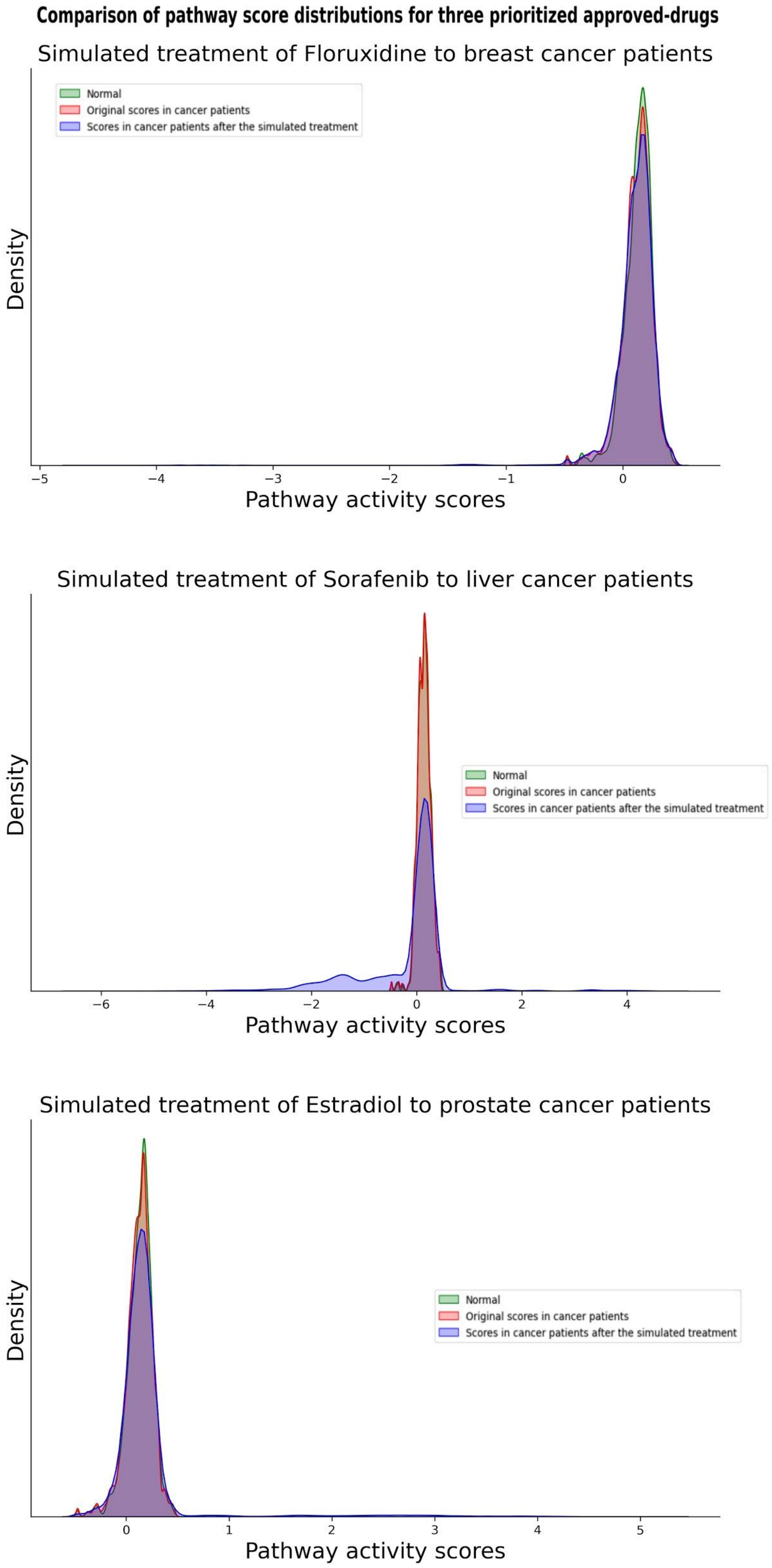

Supplementary Figure 9. Comparison of the distributions of pathway activity scores before and after the simulated treatment of three approved drugs prioritized by our approach in each cancer test dataset. In the three cases, we can see that only a minority of the pathway activity scores are modified after the simulated treatment (i.e., outliers that appear on each end of the distribution depending on the effect of the drug).

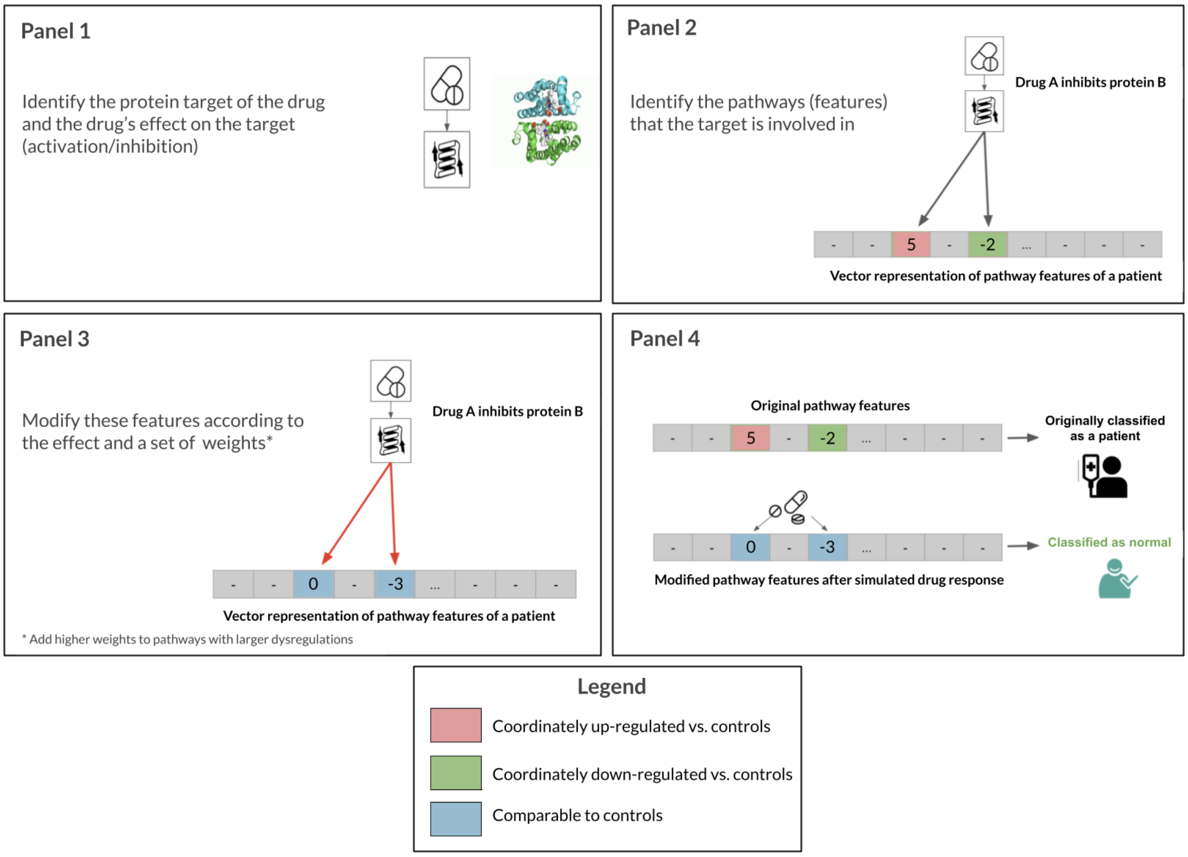

Supplementary Figure 10. Illustration of how the mechanism of action of a drug is simulated by the algorithm.

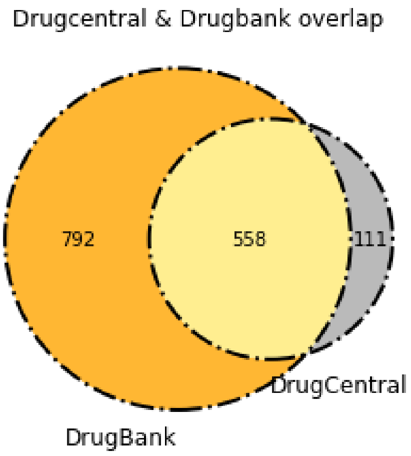

Supplementary Figure 11. Overlap of drugs present in DrugCental and DrugBank.
